# ORMDL2 Deficiency Potentiates the ORMDL3-Dependent Changes in Mast Cell Signaling

**DOI:** 10.3389/fimmu.2020.591975

**Published:** 2021-02-11

**Authors:** Viktor Bugajev, Ivana Halova, Livia Demkova, Sara Cernohouzova, Petra Vavrova, Michal Mrkacek, Pavol Utekal, Lubica Draberova, Ladislav Kuchar, Björn Schuster, Petr Draber

**Affiliations:** ^1^Department of Signal Transduction, Institute of Molecular Genetics of the Czech Academy of Sciences, Prague, Czechia; ^2^Research Unit for Rare Diseases, Department of Pediatrics and Inherited Metabolic Disorders, First Faculty of Medicine, Charles University and General University Hospital in Prague, Prague, Czechia; ^3^Czech Centre for Phenogenomics, Institute of Molecular Genetics of the Czech Academy of Sciences, Prague, Czechia

**Keywords:** mast cells, passive systemic anaphylaxis, FcϵRI, sphingolipids, ORMDL family, sphingosine-1-phosphate, passive cutaneous anaphylactic reaction

## Abstract

The systemic anaphylactic reaction is a life-threatening allergic response initiated by activated mast cells. Sphingolipids are an essential player in the development and attenuation of this response. *De novo* synthesis of sphingolipids in mammalian cells is inhibited by the family of three ORMDL proteins (ORMDL1, 2, and 3). However, the cell and tissue-specific functions of ORMDL proteins in mast cell signaling are poorly understood. This study aimed to determine cross-talk of ORMDL2 and ORMDL3 proteins in IgE-mediated responses. To this end, we prepared mice with whole-body knockout (KO) of *Ormdl2* and/or *Ormdl3* genes and studied their role in mast cell-dependent activation events *in vitro* and *in vivo*. We found that the absence of ORMDL3 in bone marrow-derived mast cells (BMMCs) increased the levels of cellular sphingolipids. Such an increase was further raised by simultaneous ORMDL2 deficiency, which alone had no effect on sphingolipid levels. Cells with double ORMDL2 and ORMDL3 KO exhibited increased intracellular levels of sphingosine-1-phosphate (S1P). Furthermore, we found that concurrent ORMDL2 and ORMDL3 deficiency increased IκB-α phosphorylation, degranulation, and production of IL-4, IL-6, and TNF-α cytokines in antigen-activated mast cells. Interestingly, the chemotaxis towards antigen was increased in all mutant cell types analyzed. Experiments *in vivo* showed that passive cutaneous anaphylaxis (PCA), which is initiated by mast cell activation, was increased only in ORMDL2,3 double KO mice, supporting our *in vitro* observations with mast cells. On the other hand, ORMDL3 KO and ORMDL2,3 double KO mice showed faster recovery from passive systemic anaphylaxis, which could be mediated by increased levels of blood S1P presented in such mice. Our findings demonstrate that *Ormdl2* deficiency potentiates the ORMDL3-dependent changes in mast cell signaling.

## Introduction

Sphingolipids constitute a branched family of lipids comprising critical structural components of cellular membranes and lipid signaling mediators in various cell types, including mast and other immune cells (1). Sphingolipids are *de novo* synthesized at the cytosolic leaflet of the endoplasmic reticulum by serine palmitoyltransferase (SPT), which condensates L-serine and CoA-activated fatty acid, preferentially palmitate, to produce 3-ketodihydrosphingosine (sphinganine). Subsequently, as a consequence of an enzymatic cascade, ceramide is formed. The ceramide serves as a central base to synthesize more complex glycosphingolipids (2–5). Besides their structural functions in the eukaryotic membranes, bioactive lipids including ceramide, ceramide-1-phosphate, sphingosine, sphingosine-1-phosphate (S1P), and lyso-sphingomyelin are involved in various cellular processes, such as migration, apoptosis, growth, senescence, adhesion, inflammation, angiogenesis, and cell signaling (3, 4).

In yeasts, Breslow et al. identified Orm1 and Orm2 endoplasmic reticulum-embedded proteins responsible for inhibiting the sphingolipid synthesis *via* the regulatory feedback pathway dependent on Orm phosphorylation (6). Mammalian orthologs of Orm proteins are ORM1-like proteins, ORMDL1, 2, and 3 (7). ORMDL proteins, which lack the phosphorylation-dependent regulatory region, use a different mechanism to inhibit SPT in the conditions of increased ceramide levels (6, 8). The tight regulation of sphingolipid biosynthesis is crucial to maintain tissue homeostasis and to prevent various disorders in development. The abnormal increase in sphingolipid synthesis seems to be behind the impossibility to prepare viable mice with combined triple knockout (KO) of all three members of the ORMDL family (9). Similarly, disruption of sphingolipid biosynthesis caused by the deletion of SPT long-chain subunit1 (SPTLC)1 or SPTLC2 caused mouse embryonic lethality (10). Furthermore, it was shown that 1) disruption of *de novo* sphingolipid synthesis in SPTLC2 heterozygous mice led to bronchial hyperreactivity in the absence of inflammation (11), 2) inducible deficiency of SPTLC2 in the intestine caused death of mice within ten days upon tamoxifen treatment (12), and 3) conditional knockout of SPTLC1 subunit in the adult bone marrow affected myeloid differentiation (13). In contrast, increased sphingolipid levels observed in the brain of ORMDL1 and ORMDL3 double KO (ORMDL1, 3 dKO) mice were linked to the defects in littermate size, disruption of proper myelination, and a neurologic phenotype (9). The sphingolipid regulator ORMDL3 was found to be also involved in calcium homeostasis (14, 15) and unfolded protein responses induced by endoplasmic stress (14, 16).

Single-nucleotide polymorphisms (SNPs) in chromosome 17q12-q21, where the human *ORMDL3* gene resides, have been associated in genome-wide association studies with the childhood onset asthma and increased ORMDL3 mRNA levels (17, 18). Recently, Ono et al. confirmed that asthma-risk genotypes in children with non-allergic asthma are connected with the lower levels of dihydroceramides (C18, C18:1, C24:1), ceramides (C18, C20, C22, C24, C24:1), and sphingomyelins (SM18, SM18:1, SM24:1) in the whole-blood cells. Interestingly, sphingolipids in the blood cells from children with high-eosinophil-count-associated asthma (allergic asthma) exhibited comparable levels of sphingolipids with control subjects, but possessed increased levels of dihydroceramide-C18 and ceramide-C20 in plasma (19). *In vivo* studies with mice lacking ORMDL3 showed contradictory results. Thus, Löser et al. demonstrated that ORMDL3 deficiency protected mice against *Alternaria*-induced allergic response (20). Furthermore, mice selectively deficient in ORMDL3 in the airway epithelium exhibited unexpected airway hyperresponsiveness associated with increased levels of S1P in the serum (21). Finally, mice with reduced or enhanced ORMDL3 levels showed a negative correlation with sphingolipid levels, but the development of experimental allergic asthma was comparable to wild-type (WT) mice (22).

Previous experiments suggested that besides ORMDL3, other members of the ORMDL family have critical biological roles. Thus, in HeLa and HEK293 cells, only silencing of all three members of the ORMDL family increased the ceramide levels (6, 23). Furthermore, ORMDL3 KO, in contrast to ORMDL1 KO or ORMDL2 KO, enhanced the sphingolipid levels in the murine brain. This enhancement was further significantly pronounced by a concurrent absence of ORMDL1 and ORMDL3 (9).

In our previous study, we prepared bone marrow-derived mast cells (BMMCs) with reduced or enhanced levels of ORMDL3 and found that ORMDL3 is a negative regulator of high-affinity IgE receptor (FcϵRI) signaling (24). We also found that BMMCs express the *Ormdl1* and *Ormdl2* genes, but their involvement in the FcϵRI activation events was not determined (24). Mast cells reside in the tissues near the external environment. They are capable of releasing granules with bioactive compounds, such as histamine and proteases, or *de novo* synthesizing eicosanoids, cytokines and signaling sphingolipids to initiate acute immune responses (25). Besides mast cell ability to produce signaling messengers such as S1P by sphingosine kinases (SPHKs) 1 and 2, they also express on their surfaces receptors recognizing them (26). These receptors can enhance or reduce the output of the FcϵRI signaling (26, 27). S1P receptor 1 (S1PR1) is important for cytoskeleton rearrangement and migration toward antigen, while S1PR2 is important for degranulation (26). SIPR2 is also important in S1P-dependent histamine clearance during passive systemic anaphylaxis (PSA) (28). The FcϵRI signaling could be attenuated by binding of extracellular ceramide to leukocyte mono-immunoglobulin-like receptor 3 (LMIR3; CD300f) *via* its two immunoreceptor tyrosine-based inhibitory motives (27).

In this study, we focused on the role of ORMDL family members 2 and 3 in mast cell signaling. We prepared whole-body ORMDL2 KO and ORMDL3 KO mice and mice with combined ORMDL2,3 deficiency (ORMDL2,3 dKO), and then we studied the properties of their mast cells and mast cell-dependent immune responses. The combined data suggest that ORMDL2 together with ORMDL3 co-ensure the proper mast cell functions.

## Materials and Methods

### Antibodies

Antibodies specific for HPRT (sc-376938), IκB-α (sc-371), p38 (sc-535), p-p38 (Tyr182; sc-7975), and SPTLC1 (sc-374143) were obtained from Santa Cruz Biotechnology. Antibodies specific for p-IκB-α (Ser32/36; #9246) and p-SYK (Tyr525/526; #2710) were purchased from Cell Signaling Technology. Antibody against SPTLC2 (ab23696) was obtained from Abcam. Horseradish peroxidase (HRP)-conjugated goat anti-mouse IgG (sc-2005) and HRP-conjugated-goat anti-rabbit IgG (sc-2004) were from Santa Cruz Biotechnology or Jackson ImmunoResearch Lab. (#115-035-003; #111-035-144). Anti-mouse FcϵRI α chain-fluorescein isothiocyanate (FITC) conjugate (Cat. No. 11-5898) and anti-mouse c-KIT (CD117)-allophycocyanin (APC) conjugate (Cat. No. 17-1171) were obtained from eBiosciences. PE-conjugated hamster anti-mouse CD29 (β1 integrin; 562801)-specific antibody was purchased from BD Biosciences. APC/Fire™ 750-conjugated anti-human/mouse β7 integrin (Cat. No. 321224)-specific antibody was purchased from BioLegend. IgE monoclonal antibody (mAb) specific for 2,4,6-trinitrophenol (TNP), clone IGEL b4 1 was produced in our laboratory from hybridoma cells obtained from Dr. A. K. Rudolph (29). SYK-specific mAb and Lyn-specific mAb were produced in our laboratory (30, 31). Specific pan-ORMDL polyclonal serum was obtained from an immunized rabbit (serum TPFIII) as previously described (24). This serum recognizes all members of the ORMDL family at least of murine, human, and rat origin. The quality of this serum in recognizing changes in the expression of ORMDL family members was confirmed elsewhere (32).

### Mice Generation

Guide (g)RNAs targeting *Ormdl2* and *Ormdl3* were designed using the gRNA selection tool CRISPOR (33). *Ormdl2*-specific gRNA (F3; the target site for Ormdl2: 5′-ACTCGAGTGATGAACAGTCG-3′) was generated using pX330 vector (34). *Ormdl3* specific gRNA was synthesized *in vitro* using linear ssDNA oligos to generate the template for *in vitro* transcription by PCR under the following conditions: initial denaturation for 1 min at 98°C, followed by 10 cycles (10 s at 98°C, 10 s at 57°C, 3 s at 72°C), 35 cycles (10 s at 98°C, 10 s at 67°C, 3 s at 72°C), and final elongation step for 3 min at 72°C using Phusion High-Fidelity DNA Polymerase and the corresponding buffer, and pX330-U6-Chimeric_BB-CBh-hSpCas9 (34) as a template. For sgRNA synthesis, a MEGAshortscript T7 kit (Ambion) was used according to the manufacturer’s instructions. Primer sequences for *Ormdl3:* F: 5′-TGTAATACGACTCACTATAGGACACGGGTGATGAACAGTCGgttttagagctagaaatagc, R: 5′-aaaagcaccgactcggtgcc-3′. The gRNAs were mixed with Cas9 mRNA in RNase-free micro-injection buffer (0.25 mM EDTA, 10 mM Tris-HCl, pH 7.4) to final concentrations of 100 ng/μl for Cas9 mRNA and 50 ng/μl for gRNAs prior to microinjection into the male pronuclei of C57BL/6 one-cell embryos. The offspring were screened by PCR-based Heteroduplex Mobility shift Assay (HMA) with primers targeting *ORMDL2* loci 5′-ATTCAGGGATTACAAGCATGAGC-3′ and 5′-GTAGGAAGATATACATTGCCTGG-3′; or *ORMDL3* loci 5′-GGCAATTGATTGCTGGTCACC-3′ and 5′-GGGAGACATGGACGATGTGC-3′, respectively. The screening PCR was performed under the following conditions: initial denaturation for 5 min, 95°C, followed by 34 cycles: 30 s, 95°C; 30 s, 59°C; 1 min, 72°C. The final elongation step lasted 3 min at 72°C. For PCR we used DreamTaq™ Hot Start Green DNA polymerase and the corresponding buffer (Thermo Scientific). The PCR products were subjected to HMA using 5% native polyacrylamide gel electrophoresis (PAGE) and separated for 45 to 60 min with a constant electric potential of 80 V. Positive founders were identified by slower migrating bands as previously described (35) and such samples were selected for Sanger sequencing. After confirming the Cas9-dependent frameshift mutation in F1 generation, mice were further backcrossed three times to C57BL/6. For routine genotyping of selected ORMDL2 KO and ORMDL3 KO mice we used PCR of tail-snip DNA with forward primers described above and reverse primers specifically based on the respective sequences obtained by Sanger sequencing. Two PCR reactions were performed to distinguish WT, KO and Hz. The following primers were used: O2-R WT; [296 bps]; 5′-GCCAGCCAGATGCCCCGACT-3′, O2-Rmut; [294 bps]; 5′-GCCAGCCAGATGCCCCCTGT-3′ O3-R WT [271 bps]; 5′-GAGCCAGATGCCGCGACTGTT-3′ and O3-Rmut [266 bps]; 5′ AGAGCCAGATGCCGCGACATC-3′. ORMDL2,3 dKO mice were generated by crossing the established ORMDL2 and ORMDL3 homozygous mutants. Mice were bred and maintained at the Institute of Molecular Genetics in a specific pathogen-free facility. All work with animals was conducted in accordance with the Institute of Molecular Genetics guidelines (permit number 12135/2010-17210) and national guidelines (2048/2004-1020).

### qPCR

RNA was isolated using MicroElute^®^ Total RNA kit (OMEGA bio-tek). M-MLV reverse transcriptase (Invitrogen) with random hexamer primers (Invitrogen) was used according to the manufacturer’s instructions to produce single-stranded cDNA. Real-time PCR amplification was performed in 10-μl reaction volumes of qPCR mix containing 1 M 1,2-propanediol, 0.2 M trehalose and SYBR green 1 (36) in 384-well plates sealed with LightCycler 480 sealing foil and analyzed by LightCycler 480 (Roche Diagnostics). The following cycling conditions were used: 3 min at 95°C, followed by 50 cycles of 10 s at 95°C, 20 s at 60°C, and 20 s at 72°C. Threshold cycle (Ct) values were determined by automated threshold analysis of the cycler. Specificity of the PCR was evaluated by examining the melting curves. HPRT and SDHA were used as reference genes, and the expression levels of mRNAs specific for members of the ORMDL family were normalized to the geometric means of the reference genes. Primers for ORMDL genes were designed according to sequencing analysis of different mutants to be negative in specific KO. The following primer sets were used for amplification of different cDNA fragments (sense/antisense; numbers in square brackets are sizes of the fragments in bps): *Hprt*, 5′-CTGGTGAAAAGGACCTCTCGAA-3′/5′-CTGAAGTACTCATTATAGTCAAGGGCAT-3′ [110]; *Sdha*, 5′-AAGGCAAATGCTGGAGAAGA-3′/5′-TGGTTCTGCATCGACTTCTG-3′ [113]; *Ormdl1*, 5′-TGGTATGTGGCTGACATATG-3′/5′-GCAAAAACACATACATCCCCAGA-3′ [134]; *Ormdl2*, 5′-CACTCGAGTGATGAACAGT-3′/5′-AGGGTCCAGACAACAGGAATG-3′ [114]; *Ormdl3*, 5′-CAACACACGGGTGATGAACAG-3′/5′-GACCCCGTAGTCCATCTGC-3′ [244].

### Cells

BMMCs were isolated after washing the bone marrow of WT, ORMDL2 KO, ORMDL3 KO, and ORMDL2,3 dKO mice with 10 ml of culture medium. The cells were cultured in RPMI-1640 medium (R8758; Sigma-Aldrich) supplemented with antibiotics (100 U/ml penicillin, 100 μg/ml streptomycin; Gibco), 71 μM 2-mercaptoethanol (2-ME), MEM non-essential amino acids, 0.7 mM sodium pyruvate, 2.5 mM l-glutamine, 12 mM D-glucose, 10% fetal bovine serum (FBS; Biosera; Cat# FB-1090/500), and 4% WEHI-3 culture supernatant as a source of IL-3 and 4% culture supernatant from Chinese hamster ovary cells transfected with the cDNA encoding murine stem cell factor (SCF), a kind gift of Dr. P. Dubreuil; INSERM, Marseille, France). BMMCs were grown for 6 to 8 weeks, tested for the presence of surface c-Kit (CD117) and FcϵRI, and then used within 2 months for the experiments.

PDMCs were isolated after washing the peritoneum of WT, ORMDL2 KO, ORMDL3 KO and ORMDL2,3 dKO mice with 15 ml of culture medium. The cells were cultured as mentioned above. PDMCs were grown for 4 weeks and subsequently tested for sphingolipids.

### Cell Activation

Cells were washed and activated in buffered saline solution (BSS; 135 mM NaCl, 5 mM KCl, 1.8 mM CaCl_2_, 5.6 mM glucose, 20 mM HEPES, pH 7.4) supplemented with 0.1% essential fatty acid-free and globulin-free bovine serum albumin (BSA; Sigma, A7030). The cells were sensitized with TNP-specific mouse IgE, clone IGEL b4 1 (29). IgE bound to FcϵRI was cross-linked with antigen (TNP-BSA conjugate, 15–25 mol TNP/mol BSA; prepared in our laboratory).

### Immunoblotting

Whole-cell extracts were prepared by washing the cells in cold phosphate-buffered saline, pH 7.2 (PBS), followed by solubilizing them in hot sodium dodecyl sulfate-(SDS)-PAGE sample buffer (37), sonicating three times for 5 s at 20°C, and boiling for 5 min. Alternatively, in experiments analyzing sphingolipids or studies aimed to quantify expression of selected proteins, 2x concentrated SDS-PAGE sample buffer was added to the aliquots of sonicated lysates. Protein extracts (25 µg) were size-fractionated in 13% SDS-PAGE gels, electrophoretically transferred onto nitrocellulose membrane (0.45 μm; GE Healthcare Life Sciences) using a Bio-rad Mini-PROTEAN apparatus. The membranes were blocked with 2% BSA-TNT solution (20 mM Tris-HCl, pH 7.5, 200 mM NaCl, 0.1% Tween) and analyzed by immunoblotting with protein-specific antibodies. Bound primary antibodies were detected with the corresponding HRP-conjugated secondary antibodies. HRP signal was detected with chemiluminescence reagent (38) and collected with Luminescent Image Analyzer LAS 3000 (FujiFilm). Aida Image Analyzer v5.0 software (Raytest) was used for signal quantification.

### Measurement of Sphingolipids

#### Extraction of Mast Cells for Analysis of Ceramides and Sphingosines

BMMCs or PDMCs (6 × 10^6^) were collected, washed in BSS-BSA buffer and then in distilled water of chromatography grade (Sigma-Aldrich). Each cell pellet was suspended in 400 µl of distilled water and sonicated three times for 5 s. One part of the sonicate was saved to detect proteins by immunoblotting. Concentration of the proteins was determined by the Bradford assay (Thermo Fisher Scientific). Samples containing 40 µg of proteins were placed into glass vials containing internal standards [C17:1 sphingosine (Cat. No. 860640P) and C17:0, d18:1 ceramide (Cat. No. 860517P) Avanti Polar Lipids]. Then, 1 ml of 2:1 chloroform/methanol solution was added. Samples were shaken horizontally for 1 h at 22°C followed by filtration through hydrophobic Millex LH 0.45 µm filters (Merck Millipore).

#### Extraction of Mast Cells for S1Ps Analysis

BMMCs (16 × 10^6^) were collected, washed in BSS-BSA buffer and then in distilled water of chromatography grade (Sigma-Aldrich). Each pellet was suspended in 200 µl of MilliQ water and homogenized by sonication for 10 s in a cup horn. Extraction of S1P was performed from 100 µl of cell homogenate by the slightly modified method of Aerts et al. (39). The entire procedure was performed in glass vials. The homogenate (100 µl) was added to a 5 ml glass tube (Chromacol 5-SV; Thermo Scientific, Langerwehe, Germany) and combined with 50 µl of MilliQ water and 100 ng of C17:1 S1P internal standard in 10 µl of chloroform/methanol (2:1, v/v) [C17:1 S1P (Cat. No. 860641P) Avanti Polar Lipids]. The first extraction step was done by addition of 900 µl of chloroform/methanol (1:2, v/v) and 15 min vortex mixing followed by 10 min centrifugation at 2500*g*. The upper phase (supernatant) was transferred to another 5 ml vial, 300 µl of chloroform and 450 µl of MilliQ water were added and 10 min vortex mixing followed. Liquid phases were separated by 5 min centrifugation at 2500*g*. The upper phase was transferred to a new 5 ml vial. The lower phase was reextracted by 1200 µl methanol/water (1:1, v/v) and 10 min vortex mixing followed by 5 min centrifugation at 2500*g*. Both upper phases were combined and evaporated under the stream of nitrogen. The extract was re-suspended in 500 µl of MilliQ water by vortex-mixing and 5 min sonication in sonication bath. S1P was then extracted by 500 µl of water-saturated 1-butanol (HPLC grade, Sigma-Aldrich) and 10 min vortex mixing followed by 5 min centrifugation at 2500*g*. Upper phase was transferred to new glass vial. Remaining lower phase was repeatedly extracted by 500 µl of water saturated 1-butanol and vortex mixing. The upper phase was separated by centrifugation and combined with the first one. Combined butanol extracts were evaporated under the stream of nitrogen. The final extract was reconstituted in 300 µl methanol and filtered through hydrophobic Millex LH 0.45 µm filters.

#### Extraction of Mouse Serum for S1P Analysis

Collected mouse sera were extracted by a slightly modified previously published method (40). The polypropylene Eppendorf tubes were replaced by 5 ml glass vials, and 100 ng of C17:1 S1P in 10 µl of chloroform/methanol (2:1, v/v) was used as internal standard. Sera were combined with PBS to achieve the total volume of 200 µl. Then, 8 µl of 1N citrate buffer with internal standard were added and twice extracted by 1-butanol. Combined butanol extracts were evaporated, re-suspended and filtered to obtain final extracts.

#### LC-ESI-MS/MS of Sphingolipids

Two separate mass spectrometry methods with the same liquid chromatography setup were used for the analysis. Ceramides were analyzed together with sphingosines by one method, whereas a separate method was used for S1P. Normal-phase HPLC was performed on a Sillica-C column 5 cm × 2.1 mm, 4 μm 100A (Cat. No. 40000-05-P-2, Microsolv Technology Corp). Isocratic elution was done in a mobile phase consisting of methanol (Biosolve) with 5 mM of ammonium acetate (LC-MS grade). The elution program for isocratic HPLC separation was as follows: flow rate 100 μl/min for 5 min. Mass spectra were measured in an AB/MDS SCIEX API 4000 tandem mass spectrometer (SCIEX: AB Sciex LLC) equipped with an ESI source and coupled to an Agilent HPLC 1290 series (Agilent Technologies). Samples were reconstituted in 300 µl of methanol with 5 mM ammonium acetate and 20 µl was introduced by the autosampler. The Analyst 1.6.3 software (AB Sciex, LLC) was used to operate the instruments and process the data.

Ceramides, sphingosines, and S1Ps were analyzed by selected reaction monitoring (SRM) in the positive ion mode *via* generating the [M+H^+^]^+^ precursor ions. The product ion used for ceramide was 264.4 m/z corresponding to C18:1 sphingoid base, whereas the sphingosine product ions in m/z were 268.3 (C17:1 internal standard), 282.3 (C18:1), 284.3 (C18:0), 310.3 (C20:1) and 312.3 (C20:1) and S1Ps product ions in m/z were 250.4 (C17:1 internal standard), 264.4 (C18:1), 284.4 (C18:0), 292.4 (C20:1) and 312.4 (C20:0). Delays between mass transitions were set to 5 ms with Q1 and Q3 operating at the unit mass resolution. The settling time was set to 500 ms for ceramides and sphingosines and no settling time was used for S1Ps. The curtain gas pressure was 20 psi. Nitrogen was used as the collision gas with the pressure set at 10 psi. The capillary spray voltage was 5.5 kV. The temperature of the nebulizing gas (nitrogen) was 200°C. The ion source gas pressure was adjusted to 20 psi for source gas 1 and 50 psi for source gas 2. The interface heater was turned on during the analysis. The ion optics settings for ceramide molecular species measurements were 50 V for the declustering potential and 10 V for the entrance potential. The collision energy was set to 36 V with a collision cell exit potential of 5 V. The dwell time for each ion transition was set to 100 ms. For sphingoid base molecular species, the ion optics setting was as follows: declustering potential was set to 40 V and entrance potential was 10 V. The collision energy was set to 17 V with a collision cell exit potential of 5.5 V. The dwell time for each ion transition was set to 300 ms. The ion optics settings for S1P molecular species measurements were 215 V for the declustering potential and 11 V for the entrance potential. The collision energy was set to 25 V with a collision cell exit potential of 5 V. The dwell time for each ion transition was set to 1000 ms.

Concentrations were calculated using a known amount of internal standards 12.5 ng for C17:0, d18:1 ceramide (MW = 551.6), and C17:1 sphingosine (MW = 285.4) and 100 ng for C17:1 S1P (MW = 365.4) added to the samples. Final concentrations were expressed as pmol of lipids per mg of proteins, volume of serum and cell counts. Molecular species (isoform) profiles were constructed using the measured signals of individual species and expressed as pmol of lipids per mg of the protein (41).

### Ca^2+^ Response and β-Glucuronidase Release

To measure the Ca^2+^ response, cells were loaded with Fura-2-AM and probenecid to a final concentration of 1 µM and 2.5 mM, respectively. After 20 min incubation at 37°C, the cells were washed in BSS-BSA/probenecid and immediately before measurement centrifuged at 805*g* at 20°C for 5 min, followed by resuspending in BSS-BSA at concentration 5 × 10^5^/ml. Aliquots of 100 µl were inserted into wells of a white 96-well plate. The cells were activated after 40 s with 100 µl of BSS-BSA supplemented with TNP-BSA (1 μg/ml). Changes in concentrations of intracellular Ca^2+^ ([Ca^2+^]_i_) were determined by spectrofluorometry using an Infinite 200M plate reader (TECAN) with excitation wavelengths at 340 and 380 nm and with constant emission at 510 nm. The activator was added manually after 30 s of basal Ca^2+^ measurements. Data are shown as the ratio em510_exc340_/em510_exc380_. β-glucuronidase release was performed as described elsewhere (24).

### Chemotaxis Assay

Chemotaxis responses were assayed in 24-well transwell chambers (SPL LIFE SCIENCES, Korea) with 8 µm-pore-size polycarbonate filters in the upper wells. TNP-specific IgE-sensitized BMMCs (0.3 ×10^6^) in 120 μl of chemotaxis medium (RPMI-1640 supplemented with 20 mM HEPES and 1% BSA) were added into the upper well, and chemoattractant (antigen, TNP-BSA; 0.25 μg/ml) in 0.6 ml of the chemotactic medium was added to the lower compartment. Cells migrating into the lower wells during the 8-h incubation period (37°C, 5% CO_2_) were counted in 50 μl aliquots using an Accuri C6 flow cytometer (BD Biosciences).

### Sucrose Density Gradient Fractionation

Sucrose density gradient separations were performed as previously described (42). Briefly, IgE-sensitized BMMCs isolated from WT or ORMDL2,3 dKO mice were used. BMMCs (15 × 10^6^) were then activated or not with antigen (TNP-BSA; 0.25 μg/ml) for 5 min. After centrifugation the cells were lysed on ice in 0.8 ml lysis buffer (10 mM Tris-HCl, pH 8.0, 50 mM NaCl, 2 mM EDTA, 10 mM glycerophosphate, 1 mM Na_3_VO_4_, 1 mM PMSF, protease inhibitor cocktail) supplemented with 1% Brij-96. The gradient was formed by adding 0.5 ml of 80% sucrose stock solution to the bottom of the polyallomer tube (13 × 51 mm; Beckman Instruments) followed by 1.5 ml of 40% sucrose containing the cell lysate, 2 ml of 30% sucrose, and 1 ml of 10% sucrose. After 4 h centrifugation at 4°C and 210,000*g*, using an SW55 Ti rotor, nine fractions were collected from the top of the gradient, and individual fractions were size-fractionated by SDS-PAGE followed by immunoblotting with PY20-HRP conjugate or LYN-specific antibody. The position of phosphorylated PAG, LYN, LAT1, and LAT2 is known from our previous studies (43, 44).

### Flow Cytometry

For analysis of FcϵRI and c-Kit, BMMCs were re-suspended in 30 µl of PBS-1% BSA containing 200-fold-diluted antibodies and stained on ice for 30 min. BMMCs for β1 integrin (CD29) or β7 integrin analysis were stained separately with PE-CD29-specific or APC/FireTM 750-β7 integrin-specific 200-fold-diluted antibodies. After washing with PBS, the cells were measured by flow cytometry. Data were acquired with BD Symphony. Mast cell populations positive for FcϵRI, c-Kit, β1 integrin, and β7 integrin were determined and further processed using FlowJo software (TreeStar).

### Bead-Based Immunoassay for Assessment of Cytokine Secretion

IgE-sensitized BMMCs (2 × 10^6^) were activated or not with 1 μg/ml of TNP-BSA for 5 h. Then, the cells were centrifuged and the supernatants (25 μl) were used for cytokine quantification using the bead-based immunoassay (LEGENDplex™ multi-analyte flow assay; mouse B effector panel 8-plex; cat No. 740820) according to the manufacturer’s instructions. Data were acquired using a BD Symphony flow cytometer equipped with 488 and 637 nm lasers. The data were analyzed using LEGENDplex™ data analysis software. IL-2, IL-12p70, IL-17A, and IFN-γ were not detected in the supernatants of non-activated or activated mast cells.

### Passive Cutaneous Anaphylaxis

Twenty µl of PBS containing 10 μg/ml of anti-TNP-specific IgE was injected intradermally into the right ear. As a control, PBS alone was injected intradermally into the left ear. After 48 h, the mice (females) were intravenously challenged with antigen (TNP-BSA; 100 μg in 200 μl PBS supplemented with 1% Evans blue/20 g mouse). Two hours later, the mice were killed and the ears removed for measurement of the amount of extravasated dye. Formamide (1 ml) was then added to each ear, which was then homogenized with the T-25 ULTRATURRAX Digital High-Speed Homogenizer Systems (IKA) and incubated at 80°C for 2 h. The samples were centrifuged at 14,000*g* for 15 min and supernatants were used for measurement of absorbance at 620 nm.

### Passive Systemic Anaphylaxis

The body temperature was measured with an accuracy of 0.1°C using a rectal thermometer from BIOSEB *in vivo* research instruments system. Monitoring of body temperature was initiated 2 weeks after acclimatization of mice (females) in the experimental room. Female mice aged 11 to 16 weeks were sensitized by intraperitoneal injection with TNP-specific IgE (45 µg in 400 µl of PBS) and 18 h later, anaphylaxis was induced by tail vein injection of antigen (TNP-BSA; 200 μg in 200 μl PBS/20 g mouse). The body temperature was recorded at 10-min intervals for at least 90 min after antigen challenge.

### Histamine Measurement

PSA was induced in mice (females) as described above, but blood was collected 90 s upon antigen injection by facial vein collection into chilled plastic tubes coated with EDTA. Plasma was centrifuged for 15 min at 4°C, 1000*g* and snap frozen in nitrogen. The assessment of histamine in the plasma was done within one month of plasma collection using an EIA kit (IM2015) according to the manufacturer’s instructions (BeckmanCoulter). Total histamine was determined from 5 × 10^5^ BMMCs according to the manufacturer’s instructions.

### Statistical Analysis

Statistical analysis was carried out using the Student’s unpaired two-tailed *t*-test for comparison of two groups. For comparison of more than three groups in the experiment, one-way or two-way ANOVA with Tukey’s or Dunnett’s *post hoc* comparison was used. Multiple comparisons were performed within the group. Calculated P values of less than 0.05 were considered significant. For each analysis, the data met the assumptions of the statistical test. Data are expressed as mean ± s.e.m. All statistical analyses were performed using GraphPad Prism version 7.3.

## Results

### Preparation of ORMDL2 KO, ORMDL3 KO, and ORMDL2,3 dKO Mice and Characterization of the ORMDL Family Members Expression in Mast Cells Isolated From These Mice

**Figure 1:**
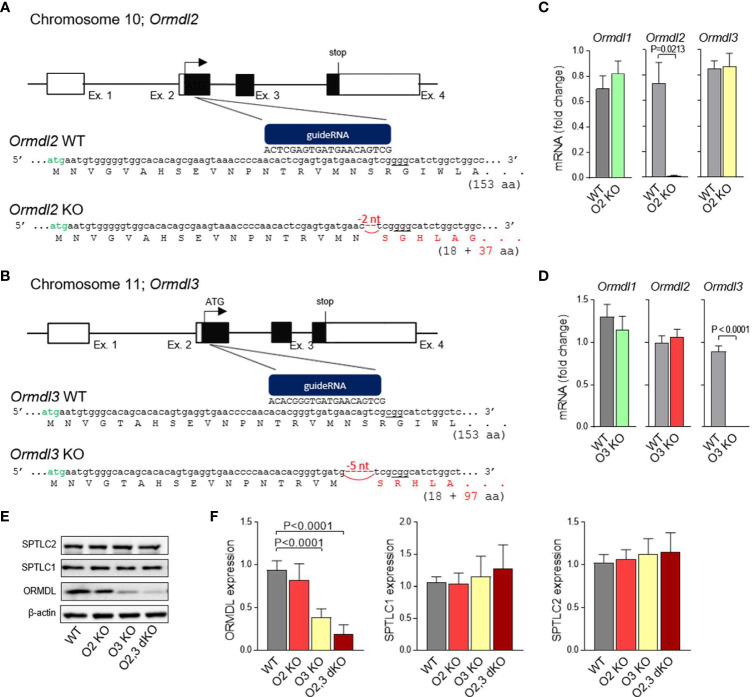
Production and initial characterization of ORMDL2 KO, ORMDL3 KO, and ORMDL2,3 dKO. **(A, B)** Strategy to generate ORMDL2 KO (O2 KO) and ORMDL3 KO (O3 KO) mice using the CRISPR/Cas9 approach. Specific guideRNAs were prepared to target the Ormdl2 and 3 within exon 2 (sites underlined) downstream of a second ATG codon (in green). Founders with 2-nucleotide or 5-nucleotide deletion leading to a frame shift in a coding sequence of the *Ormdl2* and *Ormdl3* gene, respectively, were used to establish the ORMDL2 or ORMDL3 KO mice. **(C)** RT-qPCR quantification of mRNAs encoding ORMDL1, ORMDL2, and ORMDL3 proteins in mast cells isolated from WT mice (n = 4) and O2 KO mice (n = 4). **(D)** RT-qPCR quantification of mRNAs encoding ORMDL1, ORMDL2, and ORMDL3 proteins in mast cells isolated from WT mice (n = 8) and O3 KO mice (n = 11). ORMDL2 KO and ORMDL3 KO mice were further crossed to produce ORMDL2,3 double KO (O2,3 dKO) mice. **(E)** Representative immunoblot of the lysates from BMMCs isolated from WT or O2 KO, O3 KO, and O2,3 dKO mice was developed with pan-ORMDL-specific antibody and the antibodies recognizing long chain subunits of the serine palmytoil transferase (SPT) complex. **(F)** Statistical analysis of the data presented in **(E)**; WT (n = 6), O2 KO (n = 6), O3 KO (n = 6), and O2,3 dKO (n = 6). Quantitative data are mean ± s.e.m., calculated from n, which shows numbers of biological replicates. P values were determined by unpaired two-sided Student’s t-test in **(C, D)**, or by one-way ANOVA with Tukey’s *post hoc* test in **(F)**.

ORMDL2 mRNA is expressed ubiquitously (7); however, the tissue-specific functions of ORMDL2 are unknown. To understand the role of ORMDL2 in mast cell physiology, we prepared whole-body ORMDL2 KO mice using CRISPR/Cas9 targeting the second exon of Ormdl2 resulting in a 2-nucleotide deletion at position 52-53 downstream of ATG. The hypothetical protein resulting from this prematurely terminated transcript comprises 18 amino acids of the N-terminal cytosolic part of ORMDL2 without the transmembrane domain and 37 amino acids corresponding to the new frameshift translation (**Figure 1A**). Likewise, ORMDL3 KO mice were prepared using guide RNA targeting the genomic sequence (site underlined) downstream of a second ATG codon (in green) within the first translated exon in *Ormdl3*. A founder with 5-nucleotide deletion at position 49-53 downstream of ATG was selected. The presumable non-sense transcript starts from the original ORMDL3 initiating ATG codon and terminates after 345 nucleotides (**Figure 1B**). The ORMDL2 KO and ORMDL3 KO mice were born according to the Mendelian considerations; the littermates were of standard size, developed normally and were fertile. RT-qPCR analysis of *Ormdl* gene family expression in mast cells isolated from ORMDL2 or ORMDL3 KO exhibited impaired expression of *Ormdl2* and *Ormdl3* mRNAs, respectively (**Figures 1C, D**). The expression levels of mRNAs encoding the remaining members of ORMDL were comparable with WT mast cells (**Figures 1C, D**).

To study the role of redundancy in the ORMDL family, we established ORMDL2,3 dKO mice, which were fertile and born according to the Mendelian ratios. Lysates of BMMCs from WT, ORMDL2 KO, ORMDL3 KO, and ORMDL2,3 dKO were assessed by immunoblotting using pan-ORMDL antibodies. The average decrease in ORMDL family at the protein level normalized to β-actin was ~ 18% in ORMDL2 KO, ~ 62% in ORMDL3 KO, and ~ 81% in ORMDL2,3 dKO (**Figures 1E, F**). The band of about 17 kDa observed in ORMDL2,3 dKO corresponds to the ORMDL1 expression, as the antibody used recognizes all members of the ORMDL family (24). We also examined the expression of SPTLC1 and SPTLC2, and found that the expression of subunits is similar in all cell types analyzed (**Figures 1E, F**). This excludes the possibility that the reduced expression of ORMDL2 and/or ORMDL3 family members regulates the expression of the SPT complex.

To further characterize these BMMCs, we measured their proliferation during 6 weeks of cultivation. We found that BMMCs derived from ORMDL3 KO and ORMDL2,3 dKO mice proliferated more than WT and ORMDL2 KO BMMCs (**Figure S1A**). The content of histamine in their granules and the surface expression of FcϵRI, c-Kit, and β1 integrin was comparable (**Figures S1B–D**). The expression of β7 integrin, a marker of immature mast cells (45), was not present on the surface of BMMCs 11 weeks after their isolation (**Figure S1E**). These data suggested that BMMCs derived from WT, ORMDL2 KO, ORMDL3 KO, and ORMDL2,3 dKO mice were suitable for the intended experiments.

### ORMDL2 Controls the Levels of Sphingolipids in Cells With ORMDL3 KO

**Figure 2:**
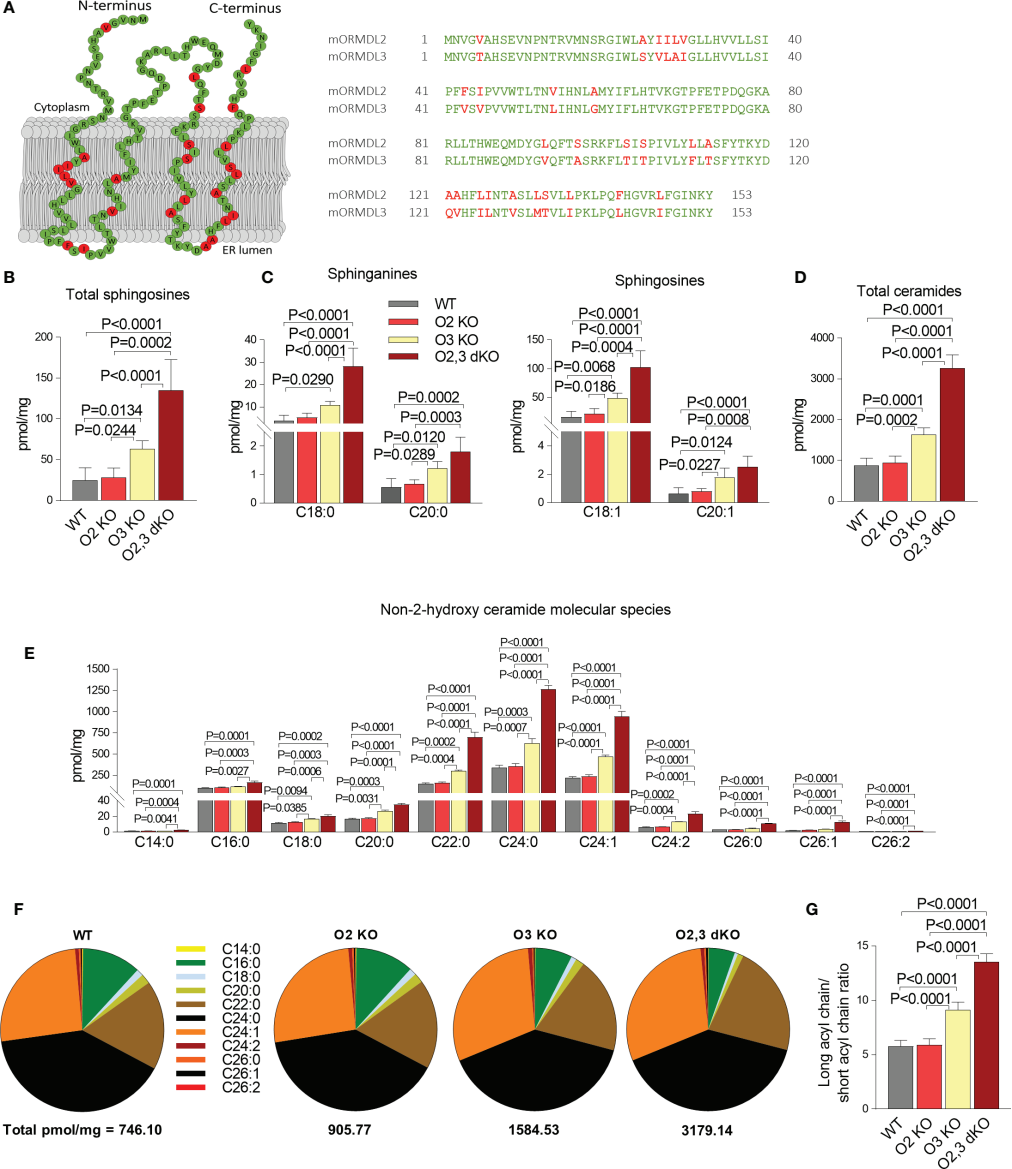
The role of ORMDL2 and ORMDL3 and their redundancy in the metabolism of sphingolipids in BMMCs. **(A)** Left: predicted topology of murine ORMDL2 based on recently described ORMDL1 topology (46). Residues marked in the murine ORMDL2 by green color indicate conserved amino acids, in red are shown residues which are different in mORMDL3. Right: alignment of primary amino acid sequences of mORMDL2 and mORMDL3. The amino acid replacements were aligned by DNASTAR Lasergene 15. **(B–E)** LC-ESI-MS/MS analysis of sphingolipids in BMMCs isolated from WT mice (n = 5), O2 KO mice (n = 6), O3 KO mice (n = 5), and O2,3 dKO mice (n = 3). **(B)** The level of total sphingosines (C18:1, C18:0, C20:1, and C20:0) is presented. **(C)** The levels of distinct sphinganines C18:0 and C20:0, and sphingosines C18:1 and C20:1 are shown. **(D)** The levels of total ceramide fatty acid chain molecular species, derived from C18:1 sphingosine. **(E)** The levels of non-2-hydroxylated ceramide isoforms derived from C18:1 sphingosine. **(F)** Proportions of individual non-2-hydroxylated ceramide isoforms. **(G)** Ratio of long acyl chains of all isoforms (C22–C26) and short acyl chains of all isoforms (C14–C20). Quantitative data are mean ± s.e.m., calculated from n, which show numbers of biological replicates. P values were determined by one-way ANOVA with Tukey’s *post hoc* test.

Proteins of the ORMDL family are highly conserved. The extent of identity between ORMDL2 and ORMDL3 at the protein level is 83% in mice and 80% in humans (7). The graphical comparison of the primary amino acid sequence of mouse ORMDL2 and ORMDL3, based on the recently determined topology of ORMDL1 by Davis et al. (46), is shown in **Figure 2A**. The difference between proteins is subtle, and most of the dissimilar amino acids are in the membrane region.

To test the hypothesis that other members of the ORMDL family sufficiently ensure the homeostasis of sphingolipid biosynthesis when ORMDL3 is missing, we used mice carrying homozygous mutant alleles of both *Ormdl2* and *Ormdl3*. Analysis of sphingolipids in BMMCs isolated from WT, ORMDL2 KO, ORMDL3 KO, and ORMDL2,3 dKO mice indicated that ORMDL2 KO alone, in contrast to ORMDL3 KO alone, has no impact on total sphingosine levels (**Figure 2B**). In BMMCs with ORMDL2,3 dKO, the overall sphingosine levels (135.0 ± 21.9 pmol/mg) were almost twice higher when compared with the sphingosine levels in BMMCs deficient in ORMDL3 alone (69.6 ± 4.5 pmol/mg; **Figure 2B**). The total sphingosines are derived either from *de novo* produced sphinganines or from the degradation of ceramides in a salvage pathway (5). Both BMMCs with ORMDL3 KO and BMMCs with ORMDL2,3 dKO exhibited increased levels of sphinganines C18:0 and C20:0 and enhanced levels of sphingosines C18:1 and C20:1 when compared with WT BMMCs and cells with ORMDL2 KO alone (**Figure 2C**). Similarly, total ceramide levels were not changed in BMMCs with ORMDL2 KO when compared with WT cells, but the ORMDL3 KO alone led to the accumulation of ceramides (1740.5 ± 75.1 pmol/mg). Even higher total ceramide levels were found in ORMDL2,3 dKO cells (3268.3 ± 184.6 pmol/mg; **Figure 2D**). The differences in distinct non-2-hydroxylated ceramide species reflected the changes in total ceramides (**Figure 2E**). It should be noted that in accordance with BMMCs, ORMDL3-deficient PDMCs exhibited increased levels of total sphingosines and ceramides when compared with WT PDMCs or ORMDL2 KO PDMCs (**Figures S2A, B**). The ORMDL2,3 dKO PDMCs showed a further increase in the levels of sphingosines and ceramides (**Figures S2A, B**). Although ORMDL2 KO in BMMCs did not affect distinct ceramide species, the combined deficiency in ORMDL2 and ORMDL3 resulted in their higher levels. The accumulation of C18:0, C20:0, C22:0, C24:0, C24:1, and C24:2 ceramides was significantly higher in ORMDL3 KO BMMCs when compared to ORMDL2 KO cells (**Figure 2E**). It should also be noted that non-2-hydroxy ceramide species with longer acyl chains were more accumulated in ORMDL2,3 dKO > ORMDL3 > ORMDL2 = WT cells (**Figures 2F, G**). When the ratio of measured ceramide species of longer (≥C22:0) and shorter acyl chains (≤ C20:0) was determined, a similar order was obtained: ORMDL2,3 dKO > ORMDL3 > ORMDL2 = WT (**Figure 2G**).

The combined data indicate that the ORMDL2-dependent decrease in ORMDL family expression (~ 19%) in ORMDL2,3 dKO mast cells (**Figure 1E**) increases the sphingolipid synthesis disproportionately.

### Increased Intracellular S1P Levels in ORMDL2,3 dKO BMMCs

**Figure 3:**
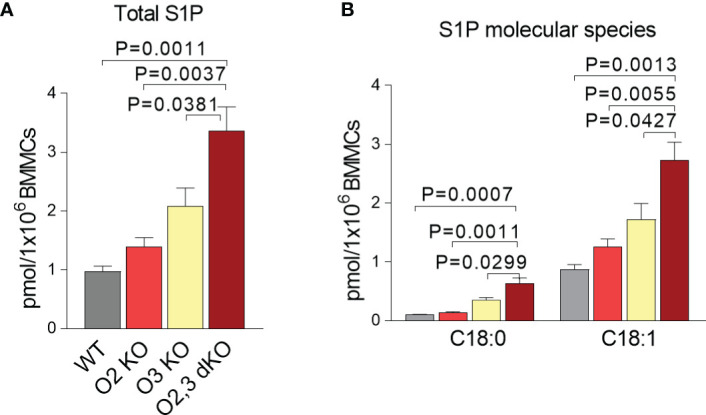
The intracellular levels of S1P in ORMDL2 and/or ORMDL3 deficient BMMCs. **(A, B)** LC-ESI-MS/MS analysis of intracellular S1P levels in BMMCs isolated from WT mice, O2 KO mice, O3 KO mice, and O2,3 dKO mice, n = 3 in all groups. **(A)** The total levels of S1P (C18:1 and C18:0) are presented. **(B)** The levels of S1P derived from sphinganine C18:0 and sphingosines C18:1 are shown. Quantitative data are mean ± s.e.m., calculated from n, which show numbers of biological replicates. P values were determined by one-way ANOVA with Tukey’s *post hoc* test.

S1P is an important signaling molecule involved in cell differentiation, proliferation, migration, angiogenesis, and survival (47). Mast cells require S1P for their stimulation (26, 47–49). We found that the intracellular levels of all S1Ps derived from C18:1 and C18:0 sphingosines were increased in ORMDL2,3 dKO when compared with ORMDL3 KO, ORMDL2 KO and WT BMMCs (**Figure 3A**). The corresponding profile was found in the S1P originated from *de novo* produced sphinganine C18:0 and in the sphingosine C18:1, which is mostly derived from the degradation of ceramides in a salvage pathway (**Figure 3B**). To determine the ratio between non-phosphorylated and phosphorylated C18:1 and C18:0 sphingosines in resting cells, we recalculated the levels of sphingosines C18:1 and C18:0 per 1 × 10^6^ of BMMCs. We found that the ratio of total sphingosines to total S1Ps was in WT = 20.4, ORMDL2 KO = 19.3, ORMDL3 KO = 28.9, and ORMDL2,3 dKO = 38.8. The ratio of C18:1 sphingosines to C18:1 S1P was comparable with the previous values. The ratio of *de novo* formed C18:0 sphinganine to C18:0 S1P was in WT = 37.9, ORMDL2 KO = 38.3, ORMDL3 KO = 30.9, and ORMDL2,3 dKO = 44.4.

### ORMDL Family-Dependent Changes in the Antigen-Mediated Calcium Mobilization, β-Glucuronidase Release and Mast Cell Migration

**Figure 4:**
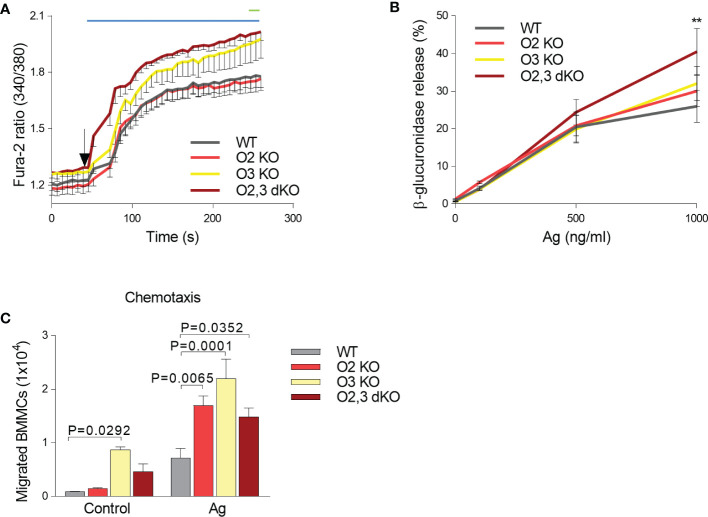
ORMDL family-dependent increase in calcium mobilization, β-glucuronidase release and migration in antigen-activated BMMCs. **(A)** Calcium response in WT (n = 8), O2 KO mice (n = 8), O3 KO mice (n = 5), and O2,3 dKO mice (n = 6). **(B)** β-glucuronidase release from non-activated and Ag-activated BMMCs isolated from WT, O2 KO, O3 KO and O2,3 dKO mice, n = 5 in all groups **(C)** Antigen-mediated chemotaxis. IgE-sensitized BMMC derived from WT, O2 KO, O3 KO, and O2,3 dKO mice were examined for their migration through 8 μm-pore-size polycarbonate filters towards antigen (TNP-BSA), n = 3 in all groups. Quantitative data are mean ± s.e.m., calculated from n, which show numbers of biological replicates. P values were determined (in **A, B**) by two-way ANOVA with Tukey’s *post hoc* test or by one-way ANOVA with Dunnett’s *post hoc* test (in **C**). In A, the range of significant differences (P < 0.05) between O2,3 dKO and WT or O2 KO cells is shown as a blue line, and between ORMDL3 KO and WT or O2 KO cells as a green line. **P < 0.01.

In our previous study, we found that ORMDL3 knockdown enhances mast cell responsiveness to antigen (24). The negative regulatory role of ORMDL3 in immunoreceptor signaling was also observed in CD4+ T cells with silenced ORMDL3, which produced enhanced levels of IL-2 after activation with antibodies to CD3 and CD28 when compared to control cells (50). In contrast, eosinophils with ORMDL3 knockdown exhibited decreased Ca^2+^ levels upon activation with eotaxin and reduced CD-48 mediated degranulation of erythropoietin (51). The known role of ORMDL family members in Ca^2+^ homeostasis (14, 15, 51) led us to compare the Ca^2+^ response of BMMCs from WT, ORMDL2 KO, ORMDL3 KO, and ORMDL2,3 dKO mice. We found that antigen-activated BMMCs with ORMDL3 KO and ORMDL2,3 dKO showed a higher Ca^2+^ response than BMMCs with ORMDL 2 KO and WT cells (**Figure 4A**). These data suggested that ORMDL3 plays a more important role than ORMDL2, which contributes to the calcium response when ORMDL3 is missing. We also tested the Ca^2+^ response of these BMMCs to SCF, but we found that all cell types examined exhibited a comparable c-Kit-mediated Ca^2+^ response (**Figure S3**).

To determine the extent of antigen-induced mast cell degranulation, we examined the β-glucuronidase release. We found that activated BMMCs with ORMDL2,3 dKO exhibited significantly increased degranulation when compared with WT BMMCs and single KOs, suggesting that the combined ORMDL2 and ORMDL3 deficiency was crucial to elevate the β-glucuronidase release (**Figure 4B**).

In our previous study we reported that chemotaxis of BMMCs with ORMDL3 knockdown was increased (24). Next, we therefore analyzed the migration of all BMMC cell types under study toward antigen. Interestingly, we found that the resting BMMCs derived from ORMDL3 KO mice exhibited increased motility leading to movement of the cells into the bottom chamber (**Figure 4C**). When the cells were exposed to antigen gradient, BMMCs deficient in ORMDL2 and/or ORMDL3 exhibited significantly increased chemotaxis when compared with WT BMMCs. Overall, these data support the hypothesis that ORMDL family members are negative regulators of mast cell signaling.

### Increased Phosphorylation of IκB-α and Cytokine Production in ORMDL2,3 dKO BMMCs

**Figure 5:**
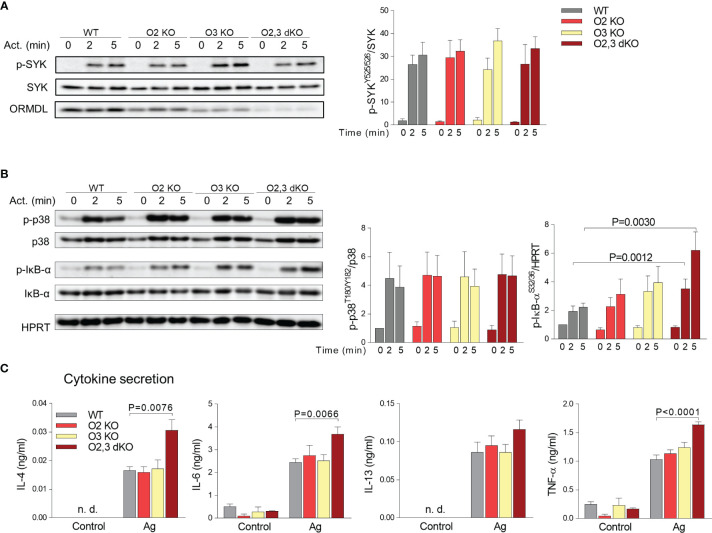
ORMDL family-dependent changes in phosphorylation of IκB-α upon FcϵRI-mediated activation of BMMCs and cytokine secretion. **(A, B)** Lysates from nonactivated or activated BMMCs were subjected to immunoblotting analysis with the indicated phospho-specific antibodies. Quantification and statistical evaluation of fold changes in protein phosphorylation were normalized to phosphorylation in non-activated cells and to the amount of the corresponding loading control (SYK, p38) or antibody specific for HPRT was used as a p-IκB-α loading control. Typical results from four to five independent experiments are presented. **(C)** The levels of IL-4, IL-6, IL-13, and TNF-α released into the supernatant from non-activated (Control) or Ag-activated (Ag) BMMCs were determined by bead-based immunoassay. WT (n = 4), O2 KO mice (n = 3), O3 KO mice (n = 4), and O2,3 dKO mice (n = 4). Quantitative data are mean ± s.e.m., calculated from n, which show numbers of biological replicates. P values in **(A–C)** were determined by one-way ANOVA with Dunnett’s *post hoc* test. n. d., Non-determined.

Having BMMCs with ORMDL2 KO and/or ORMDL3 KO, we next examined phosphorylation events associated with FcϵRI triggering by multivalent antigen. Tyrosine phosphorylation of SYK was similar in all tested groups (**Figure 5A**), suggesting that the earliest signaling events characterized by tyrosine phosphorylation of SYK were not affected by the changes in sphingolipids. Sphingolipids are enriched in the detergent-resistant membranes (DRM), which are believed to reflect the lipid raft organization of the plasma membrane (52). These lipid rafts seem to be involved in the initiation and propagation of mast cell signaling (52). We isolated the fractions of cellular lysates from WT BMMCs and ORMDL2,3 dKO BMMCs using sucrose gradient centrifugation to determine the possible enrichment of DRM-associated protein with DRM fractions (**Figure S4**). We found that the distributions of LYN kinase and tyrosine-phosphorylated adaptor proteins PAG1, LAT1 or LAT2 were comparable among the corresponding fractions originated from non-activated and antigen-activated WT and ORMDL2,3 dKO BMMCs (**Figure S4**). The phosphorylation of p38, another marker of mast cells activation, was also comparable in all tested groups (**Figure 5B**).

Previously, we also found that antigen-activated BMMCs with reduced expression of ORMDL3 exhibited enhanced activity of the NF-κB pathway (24). In further experiments, we, therefore, examined phosphorylation of the NF-κB inhibitor, IκB-α, in various BMMC types. We found that BMMCs with ORMDL2,3 dKO exhibited significantly higher phosphorylation of IκB-α when compared with other single KOs and WT BMMCs (**Figure 5B**). The increased phosphorylation of IκB-α was accompanied in ORMDL2,3 dKO BMMCs with increased secretion of interleukins (IL)-4, IL-6, and tumor necrosis factor (TNF)-α into the supernatants of antigen-activated cells (**Figure 5C**).

### Enhanced PCA in ORMDL2,3 dKO Mice

**Figure 6:**
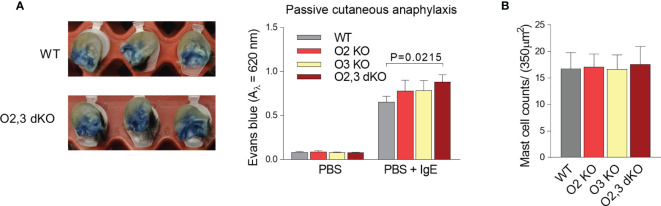
The role of ORMDL family members in PCA. **(A)** TNP-specific IgE was injected intradermally into the right ears and PBS alone into the left ears. After 48 h, TNP-BSA was administered intravenously together with Evans blue dye. Mice were killed 1 h later. The examples of excised IgE-positive ears penetrated with Evans blue from WT mice and ORMDL2,3 dKO mice are shown on the left. On the right, quantification of Evans blue extravasation from control ears (PBS) and IgE-positive ears (PBS + IgE). WT mice (n = 13), O2 KO mice (n = 6), O3 KO mice (n = 8), and O2,3 dKO mice (n = 13). **(B)** The counts of mast cells stained with toluidine blue in the skin cuts; n = 5 in all groups. Quantitative data are mean ± s.e.m., calculated from n, which show numbers of biological replicates. P values in **(A, B)** were determined by one-way ANOVA with Dunnett’s *post hoc* test.

The increased responsiveness of mast cells *in vitro*, which was mainly associated with combined deficiency in ORMDL2 and ORMDL3, led us to examine PCA. In concordance with the results mentioned above, we found that ORMDL2,3 dKO mice exhibited increased penetration of Evans blue into the ear tissue with IgE-sensitized mast cells upon intravenous injection of the corresponding antigen when compared with WT, ORMDL2 KO, and ORMDL3 KO mice (**Figure 6A**). The mast cell counts in the skin of WT, ORMDL2 KO, ORMDL3 KO, and ORMDL2,3 dKO mice were comparable (**Figure 6B**).

### Attenuation of PSA in ORMDL3 KO and ORMDL2,3 dKO Mice

**Figure 7:**
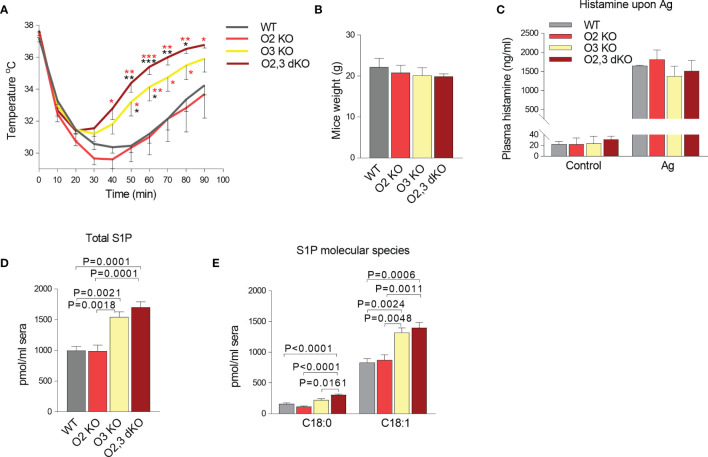
The role of ORMDL family members in the PSA. **(A)** WT mice (n = 10), O2 KO mice (n = 8), O3 KO mice (n = 10), and O2,3 dKO mice (n = 6) were sensitized i.p. with TNP-specific IgE (45 µg/mouse) and 18 h later were i.v. challenged with antigen to induce systemic anaphylaxis. Changes in body temperature were monitored at the indicated times using rectal thermometer. P values were determined by two-way ANOVA; ***P < 0.001; **P < 0.01; *P < 0.05. The significance in corresponding points is shown in black stars when compared with WT mice or red stars when compared with O2 KO mice. **(B)** Weight of mice used in the PSA experiment. **(C)** Histamine levels in WT (n = 3), O2 KO (n = 5), O3 KO (n = 5), and O2,3 dKO (n = 4) mice 90 s after antigen stimulation. **(D, E)** LC-ESI-MS/MS analysis of S1P levels in sera isolated from WT mice (n = 5), O2 KO (n = 5), O3 KO mice (n = 5), and O2,3 dKO mice (n = 5). **(D)** The total levels of S1P (C18:1 and C18:0) are presented. **(E)** The levels of S1P derived from sphinganine C18:0 and sphingosine C18:1 are shown. Quantitative data are mean ± s.e.m., calculated from n, which show numbers of biological replicates. P values in B-E were determined by one-way ANOVA with Tukey’s *post hoc* test.

Finally, we examined the role of ORMDL2 and ORMDL3 in PSA. We measured the rectal temperature drop in WT, ORMDL2 KO, ORMDL3 KO, and ORMDL2,3 dKO mice upon IgE sensitization followed by activation after antigen administration. In WT mice, the peak of decreased body temperature (30.45 ± 0.41°C) was observed 50 min after antigen administration, followed by a slow recovery, which was not completed 90 min after the challenge with antigen (**Figure 7A**). A similar course was recorded in ORMDL2 KO mice. In ORMDL3 KO mice, the maximum decrease in body temperature was lower (31.21 ± 0.48°C) and observed 30 min after antigen administration with subsequent faster recovery. In ORMD2, 3 dKO mice, the drop in the rectal temperature was maximal within 20 min, less pronounced (31.40 ± 0.60°C), and the recovery phase was the fastest, being almost completed 90 min after antigen administration (**Figure 7A**). It should be noted that the weight of mice was similar in all tested groups (**Figure 7B**). Interestingly, the levels of histamine in blood 90 s after antigen administration, was comparable among mice of distinct genotypes (**Figure 7C**). These data suggest involvement of the ORMDL family members in the recovery phase of PSA. The recovery phase of PSA is associated with the increased S1P levels (28). We therefore analyzed the S1P levels in sera from WT, ORMDL2 KO, ORMDL3 KO, and ORMDL2,3 dKO mice (**Figures 7D, E**). We found that the total levels of S1P (C18:0 and C18:1) were increased in ORMDL3 and ORMDL2,3 dKO mice when compared with WT and ORMDL2 KO mice (**Figure 7D**). A similar pattern was determined for S1P derived from *de novo* formed sphinganine C18:0 and reutilized sphingosine C18:1 (**Figure 7E**).

## Discussion

The most studied member of the ORMDL family is the abundantly expressed ORMDL3, but the presence of other two conserved members of the ORMDL family, ORMDL1 and ORMDL2, suggest involvement of an evolutionarily formed mechanism of redundancy to tightly control sphingolipid homeostasis. Indeed, the disbalance of sphingolipid synthesis in both directions could be fatal (9, 10). Recently, it has also been shown that in mammalian cells, high levels of ceramides induce ORMDL family-dependent inhibition of SPT-mediated production of sphinganines, the first intermediate in the sphingolipid *de novo* biosynthesis pathway (8).

In this study, we addressed the hypothesis that ORMDL2 could play an essential role in reducing malfunctions caused by decreased levels of ORMDL3 expression. Previously we have shown that mouse mast cells express all three genes of the family, *Ormdl1, Ormdl2, and Ormdl3*, with ORMDL3 being a dominant form as determined with pan-ORMDL antibody and by the mRNA analysis (24). We also showed that BMMCs with silenced ORMDL3 expression exhibited increased responsiveness to multivalent antigen (24). The human *ORMDL1* and *ORMDL2* genes, residing in other chromosomes than the *ORMDL3* gene, might also be associated with asthma, as peripheral blood mononuclear cells from asthmatic patients exhibited increased expression of *ORMDL1* and *ORMDL2* genes (53). To further examine the function of the ORMDL family members in mast cells and IgE-dependent signaling *in vivo*, we prepared mice with CRISPR/Cas9-mediated frameshift mutation in the *Ormdl2* or *Ormdl3* gene. By mating we also produced mice with KO of both ORMDL2 and ORMDL3. Comparison of sphingolipid levels in BMMCs isolated from WT, ORMDL2 KO, ORMDL3 KO, and ORMDL2,3 dKO mice showed that ORMDL2 deficiency caused no significant changes in the sphingolipid levels. In contrast, the lack of ORMDL3 resulted in a significant increase in all forms of measured non-phosphorylated sphingosines, including *de novo* formed sphinganines and most of the ceramide species. Importantly, the absence of both ORMDL2 and ORMDL3 resulted in a disproportional increase in the levels of all measured sphingolipids, except sphinganine C20:0 and sphingosine C20:1, when compared to the cells deficient in ORMDL3 alone. The increased C18:0 and C18:1 sphingosine levels in BMMCs with ORMDL2,3 KOs led to enhanced intracellular levels of its phosphorylated forms (S1Ps) when compared with WT, ORMDL2 KO, and ORMDL3 KO BMMCs. The mature PDMCs exhibited comparable profiles of total sphingosines and ceramides, suggesting that this phenotype is not constrained on BMMCs. These data support the concept of functional excess of ORMDL proteins relative to SPT (23) and further extend it by showing that even minor expression of ORMDL2 plays an essential role in the inhibition of sphingolipid synthesis when ORMDL3 is missing. Similarly, the dissection of ORMDL family-mediated sphingolipid synthesis in brains of mice showed that ORMDL3 is the most important member, according to an increase in sphinganine and other sphingolipid levels. At the same time, ORMDL1 or ORMDL2 deficiency did not affect the sphingolipid levels. It should be noted that in the murine brain, the function of ORMDL3 was controlled by ORMDL1 instead of ORMDL2 protein expression (9). These data suggest that the crucial role is played by the ratio of ORMDL family members in the specific tissues. The function of ORMDL1 in mast cell signaling and in the regulation of sphingolipids remains to be examined.

BMMCs isolated from ORMDL2 KO mice exhibited no significant changes in antigen-dependent signaling except migration, suggesting that minor expression of ORMDL2 plays a negligible role in the presence of ORMDL3. The chemotaxis data revealed a complex involvement of the ORMDL family in the regulation of cell motility toward antigen. The increased migration of ORMDL3 KO BMMCs confirmed our previous data (24). Although we did not found ORMDL2-dependent changes in sphingolipids, the increased migration of BMMCs deficient in ORMDL2 KO suggested that the cytoskeleton rearrangement under the plasma membrane is sensitive even to subtle changes in the membrane composition and/or S1P levels. The increased sphingolipid levels in BMMCs with ORMDL2,3 dKO or ORMDL3 KO resulted in antigen-mediated enhanced calcium mobilization when compared with ORMDL2 KO and WT cells. These data extend our previous results in which FcϵRI-induced Ca^2+^ mobilization was similar in BMMCs with ORMDL3 knockdown and BMMCs transduced with control plasmid (24). These data are in accord with the studies showing that in HEK293 and Jurkat T cell lines the silencing of ORMDL3 induces an increased Ca^2+^ response to cyclopiazonic acid, an inhibitor of sarco-endoplasmic calcium ATPase pump (14, 15). To analyze degranulation in various cell types, we measured ß-glucuronidase release. In contrast to calcium, β-glucuronidase release was enhanced in ORMDL2,3 dKO cells only. This is in line with our previous study showing comparable levels of β-glucuronidase released upon FcϵRI-mediated activation from BMMCs with ORMDL3 knockdown or control cells (24) and with the hypothesis that even minor expression of ORMDL1 and ORMDL2 set a threshold for BMMC activation when ORMDL3 is missing. Our previous findings showing that ORMDL3 knockdown increases IκB-α phosphorylation and subsequently secretion of IL-6, IL-13, and TNF-α cytokines (24) was not confirmed in this study using mice with ORMDL3 KO. On the other hand, calcium mobilization was increased in antigen-activated ORMDL3 KO BMMCs. These observed differences could be explained by using mast cells of differing origin between the studies, as we used BMMCs derived from BALB/c mice to prepare ORMDL3 knockdown (24). However, the increased responsiveness of antigen-activated BMMCs with ORMDL2,3 dKO observed in this study supports the hypothesis that the ORMDL family is involved in the negative regulation of FcϵRI signaling.

The increased proliferation of BMMCs (ORMDL3 KO = ORMDL2,3 dKO > ORMDL2 = WT), Ag-dependent Ca^2+^ mobilization (ORMDL2,3 dKO = ORMDL3 KO > ORMDL2 = WT), β-glucuronidase release (ORMDL2,3 dKO > ORMDL3 KO = ORMDL2 = WT), chemotaxis (ORMDL3 KO = ORMDL2 KO = ORMDL2,3 dKO > WT), IκB-α phosphorylation (ORMDL2,3 dKO > ORMDL3 KO = ORMDL2 = WT), secretion of IL-4, IL-6, and TNF-α (ORMDL2,3 dKO > ORMDL3 KO = ORMDL2 = WT), and PCA study (ORMDL2,3 dKO > ORMDL3 KO = ORMDL2 = WT) corroborated our previous results showing that ORMDL3 negatively regulates mast cell signaling (24). In the literature, many of the mast cell signaling events were associated with the changes in S1P levels (26, 47–49). S1P is known as a potent chemoattractant of mast cells, and the balance between S1PR expression levels is important for degranulation and motility of BMMCs (26). Phosphorylation of sphingosine to S1P is catalyzed by the activity of SPHK1 and SPHK2 (47–49). The SPHK2 isoform is a dominant producer of S1P in BMMCs, PDMCs and fetal liver-derived mast cells (48, 49). SPHK2 KO fetal liver-derived mast cells or PDMCs and BMMCs with SPHK2 knockdown exhibit decreased levels of S1P. The FcϵRI-mediated signaling in such cells exhibits reduced calcium influx, β-hexosaminidase release and expression of TNF-α, IL-6, and IL-13 cytokines (48, 49). The migration of BMMCs and PDMCs with SPHK2 knockdown toward antigen was impaired and PCA in SPHK2 KO mice was decreased (48). FcϵRI-stimulated SPHK2-deficient fetal liver mast cells exhibited defective NF-κB activation (49). We found that phosphorylation of SYK and LYN kinases associated with the initiation of FcϵRI signaling in ORMDL2,3 dKO BMMCs is comparable with WT cells, but the intracellular S1P levels are increased in ORMDL2,3-deficient mast cells. These data suggest that the enhanced S1P levels in ORMDL2,3 dKO BMMCs contribute to the increase in IκB-α phosphorylation and secretion of cytokines IL-4, IL-6, and TNF-α in antigen-activated mast cells.

The systemic anaphylactic reaction is a life-threatening allergic response accompanied by the release of histamine from antigen-activated mast cells, followed in mice by a drop in body temperature (54, 55). Previously, the authors showed that ceramides, S1P, and their receptors are potent regulators of the mast cell function *in vitro* and *in vivo* (27, 28, 56). We found that the impaired regulation of sphingolipid synthesis in ORMDL3 KO and mainly ORMDL2,3 dKO caused attenuation of antigen-dependent PSA. Interestingly, the initial step of PSA determined by histamine levels in the plasma (90 s after i.v. injection of antigen) was comparable between all examined genotypes. These data suggest that ORMDL3-deficient and ORMDL2,3-deficient mice exhibit a faster recovery phase of PSA. It should be noted that the mast cell-dependent release of histamine induces the negative regulatory feedback in PSA, based on increased production of S1P, independent of mast cells, which in turn led to the clearance of plasma histamine (28). Our finding that ORMDL3 KO and ORMDL2,3 dKO mice exhibit increased basal S1P serum levels, when compared to WT or ORMDL2 KO mice, could explain the faster PSA recovery phase in mice deficient in ORMDL3 or both ORMDL2 and ORMDL3. This suggests that ORMDL2 plays a minor role in the regulation of blood S1P levels. These data correspond to the different courses of the PSA in ORMDL3 KO and ORMDL2,3 dKO mice versus WT and ORMDL2 KO mice and could explain the discrepancy between locally developed inflammation in PCA and systemic reaction induced by PSA. The opposite course of the systemic reaction compared to the local one was reported in whole-body ORMDL3 KO mice, which exhibited attenuated airway hyperresponsiveness to *Alternaria* antigen (20), while airway epithelia-specific ORMDL3 KO had slightly increased airway responsiveness to methacholine in ovalbumin-challenged mice, presumably by enhanced S1P levels (21).

The combined data indicate that the outcome of ORMDL family member deficiency depends on several factors, including the extent of ORMDL family silencing, the tissue examined, and the *in vitro* or *in vivo* context. This suggests a complex role of sphingolipids in inflammatory responses and the crucial role of ORMDL family members in the regulation of sphingolipid levels. ORMDL2,3 deficiency caused elevation of basal S1P levels and enhanced FcϵRI-mediated responses: Ca^2+^ mobilization, β-glucuronidase release, IκB-α phosphorylation, and cytokine secretion. When analyzed *in vivo*, PCA was also increased in ORMDL2,3 dKO mice. In contrast, ORMDL3 KO and especially ORMDL2,3 dKO exhibited shortened time of the PSA recovery phase, suggesting the protective role of upregulated sphingolipid pathways in PSA.

## Data Availability Statement

The raw data supporting the conclusions of this article will be made available by the authors, without undue reservation.

## Ethics Statement

The animal study was reviewed and approved by the Animal Care and Use Committee of the Institute of Molecular Genetics.

## Author Contributions

VB, IH, and PD designed research. VB, IH, LDe, SC, PV, MM, PU, and LDr carried out the experiments. VB contributed to the production of individual KO ORMDL-gene altered mice, performed PSA, PCA, histamine quantification, β-glucuronidase assay, determined cytokine levels using bead-based cytokine assays, and prepared samples for sphingolipid measurement. IH contributed to the production of ORMDL-gene altered mice, prepared genotyping strategy and performed RT-PCR analysis. LDe performed calcium measurements, phosphorylation studies, and immunoblots. SC, PU, and MM performed phosphorylation studies and immunoblots. MM prepared drawing. PV performed immunoblots, quantification of mast cells in the skin, and assessed the growing of BMMCs. LDr performed migration assays and sucrose gradients. LK carried out mass spectrometry. BS designed the strategy for the generation of ORMDL2 KO and ORMDL3 KO mice and verified the gRNA efficiency. VB and PD wrote the manuscript and supervised the project. All authors contributed to the article and approved the submitted version.

## Funding

This work was supported by projects 17-20915S, 18-18521S and 20-16481S from the Czech Science Foundation; OP RDI CZ.1.05/2.1.00/19.0395 Higher quality and capacity for transgenic models by MEYS and ERDF; LM 2018126 Czech Centre for Phenogenomics by MEYS; OP RDE CZ.02.1.01/0.0/0.0/18_046/0015861 CCP Infrastructure Upgrade II by MEYS and ESIF; LK acknowledges the support of institutional program of the Charles University in Prague (UNCE 204064). MM was supported in part by the Faculty of Science, Charles University, Prague. LDe was supported in part by the First Faculty of Medicine, Charles University, Prague.

## Conflict of Interest

The authors declare that the research was conducted in the absence of any commercial or financial relationships that could be construed as a potential conflict of interest.

## Supplementary Material

Supplementary Figure 1 Growth properties and surface expression of FcϵRI, c-Kit, β1 integrin, and β7 integrin in BMMCs with changes in the levels of ORMDL family members. **(A)** BMMCs isolated from WT mice, O2 KO mice, O3 KO mice, and O2,3 dKO mice, n = 5 in each group, were grown for one month in the presence of IL-3 and SCF. Subsequently, 1.4 × 10^6^ of cells was transferred into fresh medium at the concentration 1.4 × 10^6^/ml. The growth of the cells was counted each week and the cells were then passaged into fresh medium at the concentration 1.4 × 10^6^/ml. **(B)** Quantification of histamine content in WT, O2 KO, O3 KO, O2,3 dKO BMMCs. **(C–E)** Flow cytometry analysis of FcϵRI, c-Kit, β1 integrin, and β7 integrin, n = 4 in each group. **(C)** Flow cytometry profile of BMMCs with surface expression of FcϵRI and c-Kit. Quantification of FcϵRI (FITC channel) and c-Kit (APC channel) is shown. **(D)** Histogram overlays of WT, O2 KO, O3 KO, and O2,3 dKO BMMCs non-labeled (−) or labeled (+) with antibody recognizing β1 integrin (in the left). Quantification of β1 integrin positive cells in PE channel (in the right). **(E)** Histogram overlays of WT, O2 KO, O3 KO, and O2,3 dKO BMMCs non-labeled (−) or labeled (+) with antibody recognizing β7 integrin. Quantitative data are mean ± s.e.m., calculated from n, which show numbers of biological replicates. P values were determined in A by two-way ANOVA with Dunnett’s *post hoc* test or by one-way ANOVA with Dunnett’s *post hoc* test **(B–D)**. ****P* < 0.001; ***P* < 0.01; **P* < 0.05.

Supplementary Figure 2 The role of ORMDL2 and ORMDL3 in the metabolism of sphingolipids in PDMCs. **(A, B)** LC-ESI-MS/MS analysis of sphingolipids in PDMCs isolated from WT mice, O2 KO mice, O3 KO mice, and O2,3 dKO mice, n = 3 in each group. **(A)** The levels of total sphingosines (C18:1 and C18:0) are presented. **(B)** The levels of total ceramide fatty acid chain molecular species, derived from C18:1 sphingosine, are presented. Quantitative data are mean ± s.e.m., calculated from n, which show numbers of biological replicates. P values were determined by one-way ANOVA with Dunnett’s *post hoc* test.

Supplementary Figure 3 The role of ORMDL family in SCF-dependent calcium signaling. Calcium response to SCF (200 ng/ml) in WT, O2 KO, O3 KO, and O2,3 dKO BMMCs, n = 5 in each group. Quantitative data are mean ± s.e.m., calculated from n, which show numbers of biological replicates. No significant intergroup differences were observed, as determined by two-way ANOVA with Dunnett’s *post hoc* test.

Supplementary Figure 4 Characterization of detergent-resistant membranes (DRM). **(A, B)** IgE-sensitized BMMCs WT and ORMDL2,3 dKO were non-activated **(A)** or activated for 5 min with antigen (TNP-BSA; 0.25 µg/ml, **B**). After solubilization in lysis buffer containing 1% Brij 96, the whole-cell lysates were fractionated by sucrose density gradient ultracentrifugation. Individual fractions were collected from the top of the gradient (fractions 1–9), size fractionated by SDS-PAGE and analyzed for tyrosine phosphoproteins by immunoblotting (IB) with PY20-HRP conjugate or with antibody specific for LYN. Positions of phosphorylated PAG, LYN, LAT1, and LAT2 are indicated by arrows on the left. Fractions (1–3) containing DRMs are also indicated. Numbers on the right indicate positions of molecular weight markers in kDa.
