# Supplementary figures and images for: ORMDL2 Deficiency Potentiates the ORMDL3-Dependent Changes in Mast Cell Signaling
Source: Front Immunol. 2021 Feb 11;11:591975. doi: 10.3389/fimmu.2020.591975 (PMC7905224; doi:10.3389/fimmu.2020.591975)

A

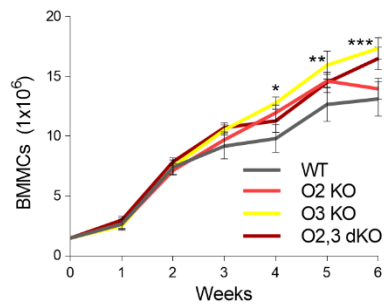

B

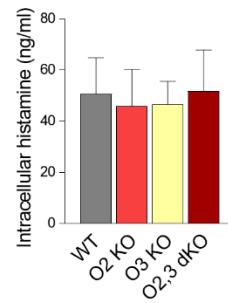

C

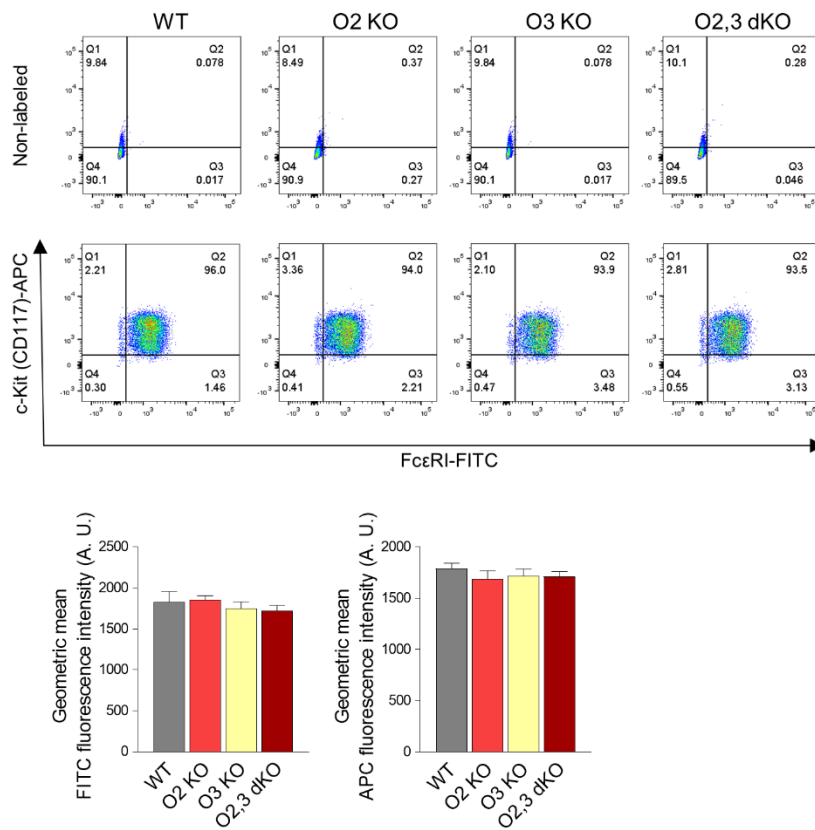

D

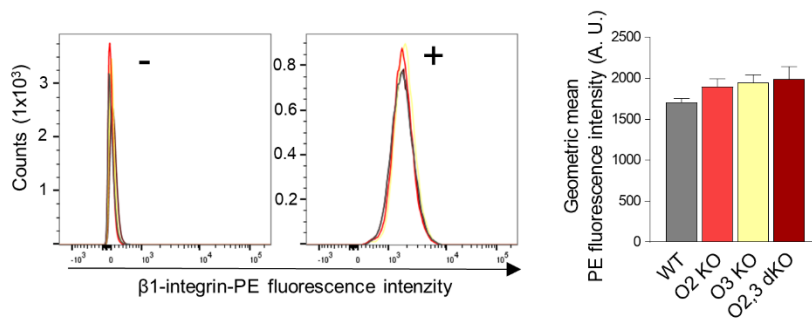

E

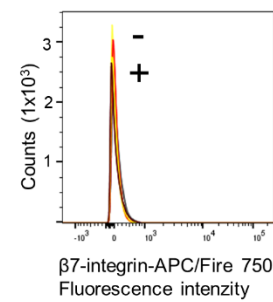

Figure S1.  
Bugajev et al.

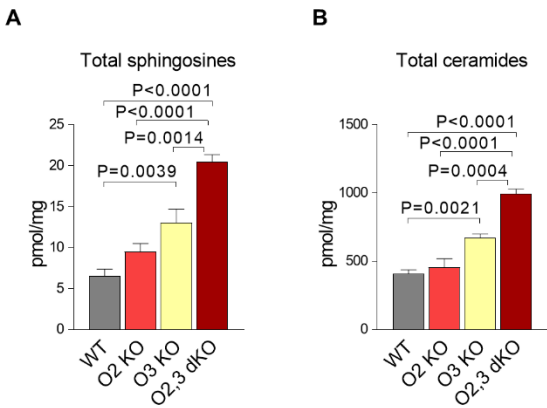

Figure S2.  
Bugajev et al.

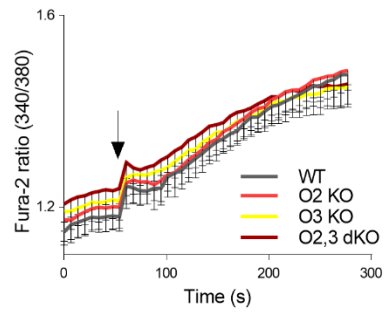

Figure S3.  
Bugajev et al.

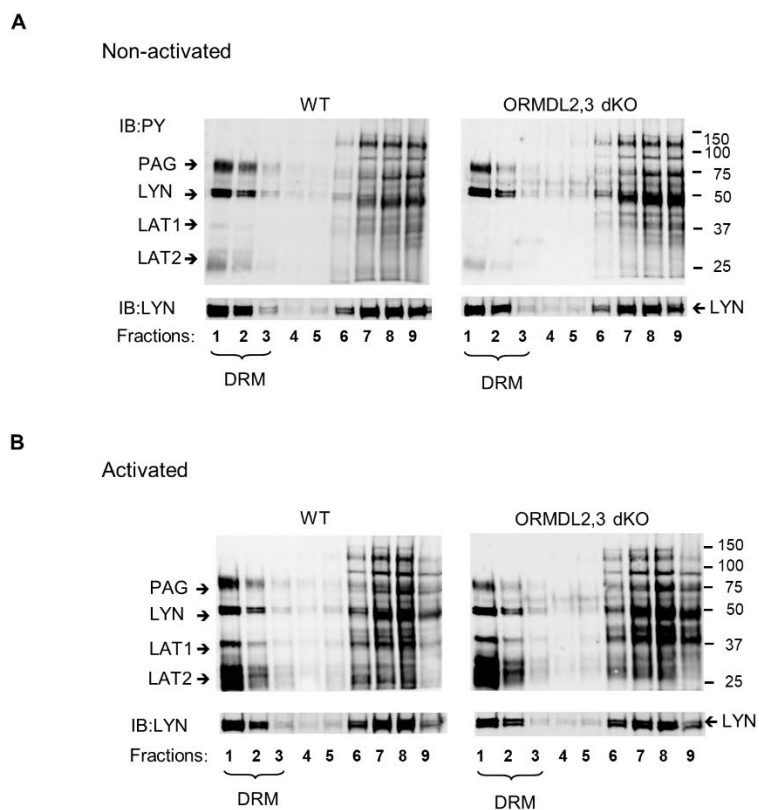

Figure S4.  
Bugajev et al.

Supplement: Supplementary Figure 1 — Growth properties and surface expression of FcϵRI, c-Kit, β1 integrin, and β7 integrin in BMMCs with changes in the levels of ORMDL family members. (A) BMMCs isolated from WT mice, O2 KO mice, O3 KO mice, and O2,3 dKO mice, n = 5 in each group, were grown for one month in the presence of IL-3 and SCF. Subsequently, 1.4 × 10^6^ of cells was transferred into fresh medium at the concentration 1.4 × 10^6^/ml. The growth of the cells was counted each week and the cells were then passaged into fresh medium at the concentration 1.4 × 10^6^/ml. (B) Quantification of histamine content in WT, O2 KO, O3 KO, O2,3 dKO BMMCs. (C–E) Flow cytometry analysis of FcϵRI, c-Kit, β1 integrin, and β7 integrin, n = 4 in each group. (C) Flow cytometry profile of BMMCs with surface expression of FcϵRI and c-Kit. Quantification of FcϵRI (FITC channel) and c-Kit (APC channel) is shown. (D) Histogram overlays of WT, O2 KO, O3 KO, and O2,3 dKO BMMCs non-labeled (−) or labeled (+) with antibody recognizing β1 integrin (in the left). Quantification of β1 integrin positive cells in PE channel (in the right). (E) Histogram overlays of WT, O2 KO, O3 KO, and O2,3 dKO BMMCs non-labeled (−) or labeled (+) with antibody recognizing β7 integrin. Quantitative data are mean ± s.e.m., calculated from n, which show numbers of biological replicates. P values were determined in A by two-way ANOVA with Dunnett’s post hoc test or by one-way ANOVA with Dunnett’s post hoc test (B–D). ***P < 0.001; **P < 0.01; *P < 0.05. [file DataSheet_1.pdf]
